# Neural Interfaces for Bidirectional Sensory Restoration in Limb Prostheses: Current Evidence and Clinical Translation

**DOI:** 10.3390/jcm15187190

**Published:** 2026-09-16

**Authors:** Fabiana Battaglia, Cristiano De Marchis, Mariarosaria Galeano, Gabriele Delia, Filippo Cucinotta, Felice Sfravara, Alexander Gardetto, Michele Rosario Colonna

**Affiliations:** 1 Department of Plastic and Reconstructive Surgery, University Hospital of Messina “AOU Gaetano Martino”, 98125 Messina, Italy; 2 Department of Engineering, University of Messina, 98166 Messina, Italyfilippo.cucinotta@unime.it (F.C.);; 3 Division of Plastic, Aesthetic and Reconstructive Surgery with Hand Surgery, Brixsana Private Clinic, Julius Durst 28, 39042 Bressanone, Italy; alexander.gardetto@brixsana.it; 4 Clinic of Plastic, Reconstructive and Aesthetic Surgery, Padova University Hospital, Via Nicolo Giustiniani 2, 35128 Padova, Italy

**Keywords:** bionic reconstruction, neural interfaces, sensory restoration, neuroprosthetics, peripheral nerve interfaces, clinical translation

## Abstract

**Background:** Restoration of sensory feedback has become one of the major goals of modern bidirectional neuroprosthetic rehabilitation, enabling the transition from conventional prosthetic replacement toward bidirectional neuroprosthetic systems capable of restoring physiological sensorimotor communication. This narrative review aims to provide a comprehensive and clinically oriented overview of current neural and related sensorimotor interfaces for upper- and lower-limb prostheses, focusing on their mechanisms of sensory restoration, clinical performance, and translational potential. **Methods:** A targeted literature search was conducted in PubMed, Scopus, and Web of Science to identify clinical, translational, and landmark studies published between January 2010 and June 30, 2026. Sources were selected according to prespecified relevance criteria consistent with the narrative design of the review. Owing to the heterogeneity of the included technologies, study designs, populations, and outcomes, the evidence was synthesized narratively. Earlier seminal publications were included when relevant to the physiological basis of sensory restoration and the historical evolution of neural interfaces. The selected literature was analyzed thematically according to neural interface technology, sensory restoration strategies, functional outcomes, and clinical translation. **Results:** Evidence from small pilot studies and experimental evaluations suggests that direct peripheral and central neural interfaces, biological or regenerative interfaces, neuromuscular signal interfaces, non-invasive sensory-feedback systems, and integrated prosthetic platforms may contribute to the restoration or substitution of tactile, proprioceptive, thermal, and multimodal sensory information. Emerging technologies, including regenerative peripheral nerve interfaces, fully implantable wireless systems, biomimetic sensory encoding, adaptive closed-loop control, and artificial intelligence-assisted decoding, may support further clinical translation, although most remain investigational. However, the available evidence remains limited by small patient cohorts, heterogeneous methodologies, and a lack of standardized long-term outcome measures. **Conclusions:** Neural and related sensorimotor interfaces have shown the feasibility of providing sensory feedback in selected experimental and early clinical settings. Although no single technology currently fulfils all clinical requirements, the integration of complementary biological, neural, and computational approaches appears to represent the most promising pathway toward personalized neuroprosthetic systems with the potential to improve sensorimotor function, although long-term clinical and quality-of-life benefits remain uncertain.

## 1. Introduction

Upper and lower limb amputation represents one of the most disabling conditions in modern medicine, profoundly affecting motor performance, sensory perception, functional independence, and quality of life. Despite substantial advances in prosthetic engineering over the past decades, restoration of natural sensorimotor function remains one of the greatest challenges in rehabilitation medicine and neuroengineering [1,2,3,4].

Modern prosthetic limbs have progressively evolved from body-powered devices to sophisticated myoelectric systems capable of reproducing increasingly complex movements. Nevertheless, although current prostheses provide improved mechanical dexterity, they still fail to reproduce the physiological bidirectional communication that exists between the peripheral nervous system and the natural limb. Consequently, most amputees continue to rely predominantly on visual feedback during object manipulation and locomotion, resulting in increased cognitive effort, reduced embodiment, impaired dexterity, and high rates of prosthesis abandonment [2,5,6,7,8].

In reconstructive surgery, “bionic reconstruction” refers to a specific multidisciplinary clinical pathway combining reconstructive procedures, advanced prosthetic fitting, and rehabilitation; in selected patients with severe and irreparable limb dysfunction, this pathway may also include elective amputation followed by prosthetic replacement. In the present review, this term is therefore reserved for this specific reconstructive pathway and is not used as a synonym for all technologies contributing to prosthetic control or sensory feedback.

The broader term “bidirectional neuroprosthetic rehabilitation” refers to the integration of motor-command acquisition with the delivery of afferent sensory information through an artificial prosthetic system. “Sensory restoration” indicates the re-establishment of sensory percepts referred to the missing limb through activation of peripheral or central neural pathways. In contrast, “sensory substitution” refers to the delivery of prosthesis-related information through an alternative intact sensory channel, without recreating the original physiological afferent pathway.

In this review, “naturalistic sensation” refers to an artificially elicited percept that approximates one or more characteristics of physiological sensation, such as its perceived location, quality, intensity, or temporal profile, without necessarily being identical to natural sensation. “Biomimetic feedback” refers to a sensory-encoding strategy designed to reproduce selected spatial, temporal, or intensity-related features of physiological sensory signaling. “Embodiment” indicates the subjective experience that a prosthesis belongs to, or is integrated into, the user’s body representation. “Clinical translation” refers to the progression of a technology from experimental feasibility toward sustained, safe, and functionally meaningful use in clinical or home settings; it is not used as a synonym for established clinical effectiveness or readiness for routine implementation.

This concept reflects the broader principles of sensory bioengineering, which aims to reproduce or substitute physiological sensory pathways through the integration of biological systems and engineered technologies, ultimately restoring functional communication between humans and artificial devices [9].

Osseointegration provides direct skeletal attachment for a prosthesis but does not, by itself, constitute a neural interface. In this review, osseointegrated systems are considered integrated prosthetic platforms when skeletal anchoring is combined with implanted muscular or neural electrodes [10,11,12,13,14,15,16].

Among these technologies, neural interfaces have emerged as the cornerstone of modern bionic prostheses. By decoding motor commands directly from peripheral nerves or reinnervated muscles while simultaneously delivering artificial sensory information through electrical stimulation, these interfaces re-establish a bidirectional communication loop that partially recreates the physiological sensorimotor pathways interrupted by limb amputation [6,10,13,14,17,18]. In this review, the term “neural interface” is used in its strict sense to indicate a technology that establishes direct recording from or stimulation of neural tissue. Technologies contributing to prosthetic control or sensory feedback without directly contacting neural tissue are described separately as biological, neuromuscular, non-invasive sensory-substitution, or integrated prosthetic approaches. The broader expression “neural and related sensorimotor interfaces” is used when referring to all these technologies collectively.

Accordingly, the technologies examined in this review are organized into six conceptually distinct categories: (1) direct peripheral neural interfaces, including extraneural and intraneural electrodes; (2) central neural interfaces involving the spinal cord, thalamus, or cerebral cortex; (3) biological or regenerative interfaces, including targeted sensory reinnervation and regenerative peripheral nerve interfaces; (4) neuromuscular signal interfaces, including targeted muscle reinnervation, agonist–antagonist myoneural interfaces, and implanted electromyographic systems; (5) non-invasive sensory-substitution systems, including transcutaneous electrical nerve stimulation, electrotactile, vibrotactile, and thermal-feedback approaches; and (6) integrated prosthetic platforms combining several components within a single system, including osseointegrated neuromusculoskeletal prostheses incorporating implanted electrodes. These categories describe different mechanisms of interaction with the sensorimotor system and should not be interpreted as interchangeable technologies.

During the last fifteen years, remarkable progress has been achieved in the development of sensory neuroprostheses. Small clinical and experimental studies have reported that tactile, proprioceptive, thermal, and force-related feedback may improve selected measures of object manipulation, grip-force modulation, embodiment, gait, and balance in selected upper- and lower-limb amputees. Recent reviews have consolidated this evidence, recognizing somatosensory feedback as a key determinant of prosthetic functionality, embodiment, and clinical acceptance [6,10,17,18,19]. More recently, fully implantable wireless systems, biomimetic sensory encoding strategies, biologically integrated neural interfaces, and artificial intelligence-based decoding algorithms may contribute to the future clinical translation of bidirectional neuroprosthetic technologies, although their readiness for routine clinical application remains to be established [13,14,17,18].

Despite growing interest in neural interfaces and bionic reconstruction, the available review articles have primarily focused on individual technologies, such as peripheral nerve interfaces, targeted muscle reinnervation (TMR), targeted sensory reinnervation (TSR), regenerative peripheral nerve interfaces (RPNIs), or sensory feedback strategies, while only a limited number have examined neural interfaces from the perspective of their clinical translation throughout the entire bionic reconstruction pathway. Given the rapid evolution of implantable neural interfaces, biological interfaces, and non-invasive sensory restoration strategies, an updated synthesis integrating current clinical evidence across both upper and lower limb applications is timely and warranted [11,14,15,16,20,21,22,23].

The distinctive contribution of this review is the integration of these conceptually different technologies within a unified clinically oriented framework that separates direct neural communication, biological and neuromuscular signal interfaces, sensory substitution, and integrated prosthetic platforms, while comparing their surgical burden, available human evidence, home-use experience, and principal translational gaps.

This narrative review aims to critically synthesize the current evidence on neural and related sensorimotor interfaces for sensory restoration and bidirectional neuroprosthetic rehabilitation in individuals with upper- and lower-limb amputation, with particular emphasis on their technological evolution, mechanisms of sensory restoration, reported functional and clinical outcomes, comparative limitations, translational challenges, and future research priorities.

## 2. Materials and Methods

### 2.1. Review Design

This study was designed as a targeted narrative review aimed at providing a clinically oriented overview of the available evidence regarding neural interfaces and related technologies for sensory restoration in upper- and lower-limb prostheses. A narrative approach was selected because of the substantial heterogeneity of the available literature in terms of interface technologies, anatomical targets, study designs, populations, sensory modalities, outcome measures, and follow-up duration. The review was not intended to constitute an exhaustive systematic review or a meta-analysis; nevertheless, a targeted literature-search and study-selection process was adopted to improve methodological transparency and reproducibility.

### 2.2. Literature Search Strategy

A targeted literature search was performed using PubMed, Scopus, and Web of Science databases to identify clinical and translational studies investigating neurosensory interfaces for bidirectional prosthetic reconstruction. The search covered publications from January 2010 to 30 June 2026, and the final database search was performed on 30 June 2026. Earlier seminal publications were additionally considered when they were necessary to describe the physiological basis, historical development, or initial clinical application of a specific technology. The search strategy combined Medical Subject Headings (MeSH) and free-text terms related to neural interfaces and bionic prostheses. The principal search terms included “neural interface”, “peripheral nerve interface”, “intraneural electrode”, “extraneural electrode”, “cuff electrode”, “TIME electrode”, “LIFE electrode”, “C-FINE”, “targeted muscle reinnervation”, “regenerative peripheral nerve interface”, “agonist-antagonist myoneural interface”, “osseointegration”, “sensory feedback”, “bidirectional prosthesis”, “bionic reconstruction”, “upper-limb amputation”, “lower-limb amputation”, “prosthetic control”, “neuroprosthetics”, and “brain–computer interface”, “spinal cord stimulation”, “epidural stimulation”, “thalamic stimulation”, “thalamic microstimulation”, “cortical stimulation”, “somatosensory cortex”, “cortical plasticity”, “thalamic plasticity”, “central neural interface”, and “central sensory restoration”.

Additional targeted searches were performed to identify studies investigating central nervous system mechanisms underlying sensory restoration, including thalamic processing, cortical plasticity, and spinal sensory stimulation, when these were considered relevant to the clinical translation of neurosensory prostheses.

The search focused primarily on studies published between 2010 and 2026, while earlier landmark publications on central and peripheral neuroplasticity were also included when considered fundamental to understanding the physiological basis of sensory restoration and the historical evolution of neural interfaces.

### 2.3. Source Selection Criteria

Priority was given to publications investigating interfaces or integrated prosthetic systems contributing to sensory restoration, sensory substitution, or bidirectional sensorimotor communication in upper- or lower-limb prosthetic applications. Human clinical studies, first-in-human investigations, pilot studies, case reports, case series, and clinically relevant translational studies were preferentially considered.

Studies primarily addressing motor decoding were considered only when the evaluated technology formed part of a bidirectional prosthetic platform or had a clearly described role in sensory restoration. Studies involving able-bodied participants or experimental animal models were considered when they represented important technological milestones or provided mechanistic evidence directly relevant to clinical translation.

Publications unrelated to limb prostheses, purely computational investigations without clinical or translational implications, conference abstracts, editorials, letters, and reports lacking sufficient methodological or clinical information were not considered central to the narrative synthesis. Peer-reviewed full conference proceedings were considered when they provided unique human neurophysiological evidence directly relevant to sensory restoration.

### 2.4. Study Selection and Study-Level Data Collection

For the studies reported in Table 1, the following information was collected, when available: study design, number and characteristics of participants, amputation level, presence of able-bodied controls, interface or device, anatomical target, sensory modality, duration of implantation or testing, study setting, comparator condition, principal outcomes, adverse events, device failures, recalibration requirements, evidence of home use, and maximum reported follow-up.

Outcome-assessment methods were classified, when reported, as objective performance tests, validated patient-reported outcome measures, study-specific experimental questionnaires, or qualitative observations. These outcome categories were interpreted separately and were not considered interchangeable indicators of clinical benefit.

Study-level information was obtained from the corresponding primary publications whenever available. Review articles were used for contextual purposes and to identify additional primary studies but were not used as the sole source for precise numerical or study-specific claims.

The information presented in Table 2 was derived from a narrative comparison of the reviewed technologies according to anatomical target, invasiveness, sensory modality, selectivity, chronic stability, hardware requirements, rehabilitation demands, documented adverse events, evidence of home use, and principal evidence gaps.

The information presented in Table 1 and Table 2 was checked against the cited publications.

### 2.5. Evidence Synthesis

Because of the substantial clinical and methodological heterogeneity of the included studies, a quantitative meta-analysis was not considered appropriate. The evidence was synthesized narratively and organized according to interface category, anatomical target, sensory modality, upper- or lower-limb application, functional outcomes, and stage of clinical development.

The synthesis distinguished between technical feasibility, elicitation of an experimentally measurable sensory percept, improvement in laboratory task performance, sustained home use, patient-reported benefit, clinically meaningful improvement in activities of daily living, and readiness for routine clinical implementation.

### 2.6. Methodological Quality Appraisal

Methodological quality was appraised qualitatively according to study design, considering participant selection, presence of a comparator or control condition, blinding, repeated-measures design, potential learning effects, duration and completeness of follow-up, reliability of outcome assessment, and selective outcome reporting. Case reports, case series, and non-randomized experimental studies were interpreted in accordance with the relevant domains of the JBI critical appraisal tools. No aggregate quality score was calculated because of the substantial heterogeneity of the included study designs.

Publications from the same research groups were also examined for potentially overlapping participants or sequential experiments. When overlapping cohorts were identified or could not be excluded, the corresponding publications were interpreted as related reports rather than as independent clinical evidence.

## 3. Results 

### 3.1. Clinical Translation of Neural and Related Sensorimotor Interfaces in Limb Prostheses

Although significant progress in neural engineering has been achieved over the past two decades, only a limited number of neural interface technologies have successfully progressed from experimental laboratory research to clinical application. The translation of these systems into routine clinical practice requires not only high-quality neural signal acquisition and selective sensory stimulation, but also long-term biocompatibility, surgical feasibility, functional reliability, patient safety, and meaningful improvements in daily activities. [1,2,3,4,5,6,7,9,10,11,12,13,14,15,16,17,18,20,23,25,31,34,37,40,41,42,43].

Clinical translation has therefore emerged as the principal benchmark for evaluating modern neuroprosthetic systems. Recent reviews have further emphasized that long-term stability, patient-centered functional outcomes, and scalable clinical implementation remain the principal challenges for the widespread adoption of somatosensory neuroprostheses [44].

While numerous interface designs have demonstrated promising experimental performance, relatively few have been investigated in human amputees through prospective clinical studies. The available evidence indicates that neural interfaces can elicit measurable sensory percepts and may improve selected motor, sensory, and embodiment outcomes under experimental conditions; however, evidence of consistent quality-of-life benefits or superiority over conventional myoelectric prostheses remains insufficient. However, important differences remain among the various technological approaches regarding invasiveness, durability, sensory selectivity, and readiness for widespread clinical implementation [8,10,12,25].

The following sections summarize the principal clinical evidence currently available for upper and lower limb bionic prostheses, highlighting the strengths and limitations of each neural interface technology.

### 3.2. Evolution of Clinical Translation

The clinical evolution of neural interfaces for bionic reconstruction has been characterized by a progressive transition from experimental proof-of-concept studies toward early-stage neuroprosthetic systems undergoing clinical evaluation capable of restoring bidirectional communication between the human nervous system and artificial limbs. While early investigations primarily focused on demonstrating the technical feasibility of recording motor commands and delivering artificial sensory information through peripheral nerve stimulation, more recent research has progressively shifted toward improving long-term stability, functional performance, patient embodiment, and real-world usability [5,6,7,8,28,45].

The functional evolution of these technologies can be illustrated through several representative milestones. Early studies, such as Rossini et al. (2010) and Raspopovic et al. (2014), primarily demonstrated the feasibility of motor decoding and experimentally elicited sensory percepts in single participants [5,6]. Subsequent investigations evaluated biomimetic feedback during controlled functional tasks and extended implantation periods, including the six-month assessment reported by Petrini et al. (2019) [26]. Later studies examined sustained use outside short laboratory sessions, including home use of osseointegrated neuromusculoskeletal prostheses over 3–7 years and use of an implanted lower-limb sensory neuroprosthesis over 31 weeks. More recently, fully implanted wireless systems have demonstrated the feasibility of integrating motor control and sensory stimulation without external percutaneous connections. This progression reflects a transition from percept elicitation and laboratory proof of concept toward integrated systems designed for prolonged use, although routine clinical effectiveness remains unestablished.

The earliest generation of implantable neural interfaces demonstrated that peripheral nerves could serve simultaneously as a source of motor information and as a target for artificial sensory stimulation. These pioneering investigations represented a major conceptual breakthrough, proving that robotic prostheses could establish direct communication with the nervous system rather than functioning as simple mechanical replacements. In particular, the introduction of longitudinal intrafascicular electrodes (tf-LIFEs) and, subsequently, transverse intrafascicular multichannel electrodes (TIMEs), provided the first evidence that selective intraneural stimulation could evoke localized tactile sensations while improving voluntary prosthetic control and object manipulation [5,6].

Following these initial demonstrations, research progressively moved beyond simple feasibility toward the evaluation of laboratory functional and patient-reported outcomes. Objective laboratory tests assessed measures such as manual dexterity, grip-force modulation, object discrimination, and task performance. Embodiment, confidence, perceived prosthesis weight, and usability were generally evaluated through validated patient-reported measures, study-specific experimental questionnaires, or qualitative observations, depending on the study. Prosthesis acceptance, functional independence, activities of daily living, and quality of life were less consistently evaluated and should not be inferred from improvements in laboratory performance or embodiment alone. Rather than assessing only signal acquisition or sensory perception, subsequent studies increasingly focused on manual dexterity, grip-force modulation, embodiment, prosthesis acceptance, and performance during activities of daily living. This transition marked an important conceptual change in neuroprosthetic research, reflecting the recognition that successful clinical translation depends not only on engineering performance but also on meaningful improvements in patient function and quality of life [28,45].

As summarized in Table 1, the field has subsequently diversified into several complementary technological approaches.

Intraneural interfaces have demonstrated high spatial selectivity for sensory stimulation in small experimental cohorts, whereas biological interfaces, including regenerative peripheral nerve interfaces (RPNIs), have introduced a regenerative strategy that combines stable electromyographic recording with meaningful sensory restoration. At the same time, extraneural cuff electrodes, osseointegrated neuromusculoskeletal prostheses, epidural spinal stimulation, and non-invasive electrotactile stimulation have considerably expanded the spectrum of clinically available solutions, each offering distinct advantages in terms of invasiveness, surgical complexity, long-term stability, and functional performance [5,7,8,10,12,17,18,24,26,27,28,29,30,31,33,34,35,36,37,38,39,40].

Another important milestone in the evolution of neural interfaces has been the progressive extension of clinical follow-up. While the earliest studies were primarily limited to acute intraoperative evaluations or short-term laboratory testing, recent investigations have demonstrated the feasibility of maintaining stable bidirectional communication over prolonged periods, including continuous home use. Long-term studies on osseointegrated neuromusculoskeletal prostheses and regenerative peripheral nerve interfaces have shown that reliable neural recordings and sensory restoration can be maintained for months or even years, supporting sustained prosthetic control in everyday life [8,10,12]. These findings support longer-term feasibility in selected participants but do not establish readiness for routine clinical adoption.

Concurrently, the objectives of neuroprosthetic research have progressively expanded beyond the simple restoration of tactile perception. Current investigations increasingly focus on recreating multiple sensory modalities, including proprioception, thermal discrimination, and somatotopic sensory perception. Reported outcomes include objective measures of laboratory task or locomotor performance, experimentally assessed sensory percepts, and patient-reported measures of embodiment or phantom-limb experience. Evidence of improved functional independence remains limited. This paradigm shift reflects the growing recognition that successful bionic reconstruction should restore the entire sensorimotor loop rather than motor function alone [10,17,37,40].

Despite these encouraging advances, several barriers continue to limit widespread clinical implementation. As highlighted in Table 1, most available evidence derives from pilot clinical studies involving very small patient cohorts, often with heterogeneous study designs, variable rehabilitation protocols, and different outcome measures, making direct comparison between technologies particularly challenging. Moreover, the absence of standardized clinical endpoints and the limited availability of long-term multicenter studies currently prevent definitive conclusions regarding the superiority of individual neural interface strategies [20].

Overall, the available evidence suggests a progressive transition from isolated proof-of-concept studies toward clinically oriented neuroprosthetic systems designed to provide increasingly stable and intuitive bidirectional communication between the user and the prosthetic limb. Importantly, this evolution has not only expanded the number of available neural interface technologies but has also progressively broadened the range of sensory and motor functions investigated. Consequently, the challenge has expanded beyond feasibility to include chronic reliability, scalability, and real-world clinical effectiveness. The following sections therefore focus on the functional outcomes achieved through sensory restoration and on the comparative advantages and limitations of the principal neural interface technologies currently available for clinical translation.

### 3.3. Sensory Restoration and Functional Outcomes

#### 3.3.1. Upper-Limb Application

Upper-limb sensory-feedback systems primarily aim to improve grip-force regulation, object discrimination, manual dexterity, and reduction of visual dependence. The most frequently investigated peripheral targets include the median, ulnar, and radial nerves, while outcomes are generally assessed through sensory-discrimination and controlled object-manipulation tasks.

The restoration of sensory feedback represents the cornerstone of modern bidirectional neuroprosthetic systems and constitutes one of the principal factors distinguishing contemporary bionic reconstruction from conventional myoelectric prostheses. Recent reviews have emphasized that restoration of sensory information is fundamental not only for improving motor performance but also for promoting embodiment, cognitive integration, and long-term acceptance of bionic hands [46]. Although current commercial prosthetic devices have achieved remarkable advances in motor control, the absence of physiological afferent feedback continues to represent one of their major limitations. Consequently, amputees frequently rely on continuous visual monitoring to regulate grasping force, manipulate objects, and coordinate limb movements, resulting in increased cognitive workload, slower motor execution, reduced embodiment, and limited functional performance during activities of daily living [6,20,25,47].

The first generation of clinical studies primarily focused on restoring tactile perception through direct stimulation of peripheral nerves. These pioneering investigations demonstrated that selective activation of intraneural electrodes could evoke localized sensations referred to specific regions of the phantom limb, allowing users to perceive contact, pressure, and object interaction in real time. Beyond demonstrating technical feasibility, these studies provided the first evidence that artificial tactile information could be successfully integrated into voluntary motor control, thereby establishing the basis for closed-loop neuroprosthetic systems [5,6].

A major advance in sensory encoding was provided by Graczyk et al., who demonstrated that the perceived intensity of artificial tactile sensations can be systematically modulated by varying stimulation pulse width and pulse frequency. They introduced the concept of Activation Charge Rate (ACR), showing that sensory intensity can be predictably controlled and reliably matched to natural tactile stimuli, thereby providing an important neurophysiological basis for biomimetic sensory encoding in closed-loop neuroprosthetic systems [48].

Subsequent clinical investigations confirmed that tactile restoration significantly improves grip-force regulation, object discrimination, manipulation accuracy, and manual dexterity while simultaneously reducing visual dependence during grasping tasks [25,28]. These findings have been consistently supported by recent reviews, which recognize tactile feedback as one of the most influential determinants of prosthetic acceptance and functional performance in upper-limb amputees [23].

However, tactile sensation alone is insufficient to reproduce the complexity of physiological sensorimotor integration. In the intact nervous system, proprioceptive information continuously informs the central nervous system about limb position, joint movement, and muscle contraction, enabling precise motor planning without the need for visual control. Restoring this sensory modality has therefore become one of the major objectives of next generation neuroprosthetic systems [49]. Clinical studies employing regenerative peripheral nerve interfaces, intraneural stimulation, and advanced sensory encoding strategies have demonstrated that meaningful proprioceptive percepts can be elicited in amputees, resulting in improved movement accuracy, smoother motor execution, greater confidence during prosthetic use, and enhanced embodiment [10,17]. Recent reviews further suggest that the combination of tactile and proprioceptive feedback may more closely reproduce natural sensorimotor integration than either modality alone, representing an important step toward physiologically biomimetic prosthetic control [47,50,51].

Another emerging area concerns multimodal sensory restoration. Rather than delivering a single sensory cue, contemporary neuroprosthetic systems increasingly combine tactile, proprioceptive, force-related, and thermal information within integrated bidirectional platforms. One of the most innovative developments has been the restoration of thermal discrimination through sensorized prosthetic hands, demonstrating for the first time that amputees can distinguish objects according to temperature using an artificial limb. Similarly, recent electrotactile and neuromorphic encoding strategies have shown that multiple sensory variables—including grasp force, object stiffness, hand aperture, and rotational movement—can be simultaneously transmitted to the user, generating increasingly natural perceptual experiences [35,52,53]. These advances indicate that future neuroprosthetic systems will likely rely on multimodal sensory integration rather than isolated feedback channels.

The evolution of implantable neurotechnologies is progressively shifting from conventional open-loop systems toward adaptive closed-loop architectures capable of continuously recording neural activity, decoding physiological biomarkers, and dynamically adjusting stimulation parameters in real time. This strategy has the potential to improve long-term stability, reduce unnecessary stimulation, optimize energy consumption, and enhance functional performance through patient-specific adaptation [54,55].

In addition to objective laboratory outcomes, selected studies have reported changes in embodiment and neurocognitive adaptation using patient-reported questionnaires or experimental assessments. The perception that the prosthetic limb belongs to one’s own body is increasingly recognized as a fundamental determinant of long-term prosthesis acceptance. Selected small studies have reported that realistic sensory feedback may promote prosthesis embodiment, reduce cognitive effort during experimental motor tasks, and improve user confidence; however, consistent long-term quality-of-life benefits have not been established [56]. Experimental studies further suggest that restoration of physiological afferent input facilitates cortical reorganization and adaptive neuroplasticity, supporting a more natural integration of the artificial limb within existing sensorimotor networks [8,10,25,57,58].

#### 3.3.2. Lower-Limb Applications

Lower-limb systems have different clinical objectives, including restoration of plantar contact and joint-position information, improvement of weight transfer, gait symmetry, balance, walking confidence, and reduction of visual dependence during locomotion. Investigated targets include the tibial and other residual lower-limb sensory pathways, while outcomes are generally assessed using gait analysis, balance measures, metabolic cost, patient-reported walking confidence, and evidence of sustained use during ambulation.

In the experimental study by Preatoni et al., patient-reported assessments indicated an approximately 23% reduction in perceived prosthesis heaviness, together with improvements in reported embodiment and confidence during walking. These findings suggest that restoring physiologically meaningful sensory input not only enhances functional performance but also facilitates incorporation of the prosthetic limb into the body schema, ultimately improving the subjective experience of prosthesis use [59].

In lower-limb applications, the reported effects of sensory feedback extend beyond sensory-percept elicitation itself. Several studies have reported reductions in phantom limb pain, improvements in gait symmetry, enhanced postural stability, and greater confidence during ambulation following restoration of somatosensory feedback [6,10,17]. Although the mechanisms underlying these effects remain incompletely understood, it has been proposed that restoration of coherent afferent input may reduce maladaptive cortical plasticity associated with long-term deafferentation, thereby contributing to both functional recovery and pain modulation [60,61,62].

#### 3.3.3. Cross-Cutting Considerations

Across upper- and lower-limb applications, preliminary evidence suggests that different interface technologies address distinct components of sensory and sensorimotor function. Intraneural electrodes have demonstrated high sensory selectivity and biomimetic percepts in selected experimental studies, biological interfaces demonstrate encouraging long-term stability while exploiting regenerative mechanisms, whereas non-invasive technologies offer a favorable balance between safety, accessibility, and functional benefit despite a lower degree of sensory specificity. Collectively, these findings suggest that future developments should not focus on replacing one technology with another but rather on integrating complementary sensory modalities capable of recreating increasingly complete sensorimotor loops tailored to individual patient needs [20,50].

### 3.4. Central Neurosensory Processing: Thalamic and Cortical Mechanisms Underlying Sensory Restoration

Although modern neural interfaces restore sensory information through stimulation of peripheral nerves or biological interfaces, the perception of these artificial sensations ultimately depends on processing within the central nervous system. Sensory information generated by neurosensors is transmitted through ascending somatosensory pathways to the thalamus and subsequently integrated within primary and secondary somatosensory cortical areas where tactile perception, proprioception, embodiment, and motor planning are generated. Consequently, successful clinical translation depends not only on the performance of the neural interface itself but also on the capacity of the central nervous system to reorganize and integrate artificial sensory inputs [63,64].

From a clinical perspective, central adaptation may influence candidate selection, sensory-encoding strategies, rehabilitation requirements, and outcome assessment. Cognitive status, duration of deafferentation, phantom-limb phenomena, residual sensory pathways, and the ability to participate in repeated training may affect how artificial feedback is interpreted and incorporated into prosthesis control. Biomimetic and somatotopically congruent encoding may facilitate adaptation, whereas less intuitive sensory-substitution strategies may require longer and individualized training. Accordingly, outcomes should be assessed not only immediately after stimulation but also after rehabilitation and repeated use, including percept stability, recalibration requirements, embodiment, functional performance, patient-reported benefit, and retention during home use.

Direct central stimulation represents a distinct interface strategy and should be distinguished from central responses induced indirectly by peripheral or non-invasive stimulation. One of the earliest demonstrations of central sensory restoration was provided by Davis et al., who showed that direct microstimulation of the human somatosensory thalamus could evoke localized phantom limb sensations closely resembling those experienced before amputation. This landmark study established that meaningful sensory percepts may be generated not only through peripheral nerve stimulation but also through direct activation of central somatosensory pathways, highlighting the pivotal role of the thalamus in sensory integration. Subsequent investigations demonstrated that limb amputation induces both immediate and long-term thalamic plasticity, with progressive reorganization of thalamic receptive fields contributing to phantom sensations and influencing subsequent cortical remapping. More recently, abnormal gamma oscillatory activity has also been identified within the somatosensory thalamus of amputees, further supporting the concept that central neural reorganization represents a fundamental component of post-amputation sensory processing [64,65,66].

In contrast to direct central stimulation, neuroimaging and neurophysiological studies of peripheral and non-invasive interfaces evaluate the central responses associated with sensory input delivered elsewhere in the nervous system. These studies suggest that restoration of peripheral sensory input may promote adaptive cortical reorganization. Functional neuroimaging, electrophysiological recordings, and magnetoencephalographic studies have demonstrated that realistic tactile feedback progressively reshapes sensorimotor cortical representations, improves embodiment, facilitates motor planning, and enhances integration of the prosthetic limb within the body schema. In particular, selective sensory stimulation has been shown to improve movement decoding while simultaneously enhancing phantom limb perception, suggesting that cortical adaptation directly contributes to the functional performance of modern neuroprosthetic systems. Similarly, studies investigating cortical remapping following targeted sensory reinnervation have demonstrated that restoration of afferent input partially reverses maladaptive cortical reorganization induced by deafferentation, supporting a more physiological sensorimotor organization [15,67,68,69].

Additional neurophysiological investigations have further clarified the mechanisms underlying this adaptive process. Selective intraneural stimulation has been shown to generate reproducible cortical somatosensory evoked potentials, confirming that artificial sensory information delivered through peripheral neurosensors reaches higher cortical centers through physiological ascending pathways. Magnetoencephalographic studies have further demonstrated cortical activation following transcutaneous electrical nerve stimulation and tactile feedback delivered by myoelectric prostheses, while selective peripheral nerve stimulation has been shown to enhance sensorimotor integration within the primary motor cortex. Collectively, these findings show that both peripheral invasive and non-invasive stimulation can produce measurable cortical responses. However, such neurophysiological responses should be interpreted as evidence of central processing and not, by themselves, as evidence of clinically meaningful sensory restoration or long-term prosthetic benefit [70,71,72,73].

Overall, the available evidence suggests that successful sensory restoration should no longer be interpreted as the simple consequence of peripheral neural stimulation. Rather, the effectiveness of modern neurosensors depends on the continuous interaction between peripheral neural interfaces, ascending sensory pathways, thalamic processing, and adaptive cortical plasticity. Future bidirectional neuroprosthetic systems will therefore likely require simultaneous optimization of peripheral interface technology, biomimetic sensory encoding, and individualized rehabilitation to facilitate central adaptation. The clinical relevance of this adaptation should be evaluated through percept stability, functional outcomes, patient-reported benefit, and sustained prosthesis use rather than through neurophysiological responses alone [23,47,74].

### 3.5. Comparative Analysis of Current Neural Interface Technologies

The rapid evolution of neural interface technologies has generated a wide spectrum of approaches for restoring bidirectional communication between amputees and prosthetic devices. However, despite remarkable technological advances, no currently available interface simultaneously achieves the ideal combination of high sensory selectivity, long-term stability, minimal invasiveness, surgical simplicity, and widespread clinical applicability. Achieving high spatial selectivity while preserving long-term biocompatibility remains one of the principal engineering challenges in peripheral neural interfaces, since increasing recording specificity is often associated with greater invasiveness and surgical complexity [75,76]. Rather than identifying a universally superior technology, the available evidence suggests that each neural interface occupies a distinct position along the translational pathway, with specific advantages and limitations that should be considered according to the clinical scenario, level of amputation, and functional objectives.

Direct comparison across technologies remains limited by differences in participant population, prosthetic system, stimulation paradigm, rehabilitation protocol, follow-up duration, and outcome measures. The characteristics summarized in Table 2 should therefore be interpreted as technology-specific considerations rather than as evidence of comparative superiority.

This variability reflects a fundamental engineering principle whereby improvements in recording selectivity and signal quality are generally accompanied by increased invasiveness, greater surgical complexity, and reduced anatomical coverage. Consequently, the optimal neural interface should be selected according to the intended clinical application rather than technological performance alone [41]. Intraneural interfaces have demonstrated high spatial selectivity for peripheral sensory stimulation in small experimental studies; however, direct comparative evidence against other interface types remains limited. By establishing direct contact with individual nerve fascicles, devices such as TIMEs, LIFEs, and USEAs can elicit localized tactile percepts with high spatial resolution while simultaneously providing accurate motor decoding.

Overall, peripheral neural interfaces should be regarded as a continuum balancing invasiveness, neural selectivity, chronic stability, and surgical complexity rather than as distinct technological categories. Extraneural cuff electrodes provide excellent long-term stability with limited spatial selectivity, whereas intrafascicular electrodes achieve greater recording specificity at the expense of increased implantation complexity. Regenerative interfaces may provide anatomically specific signal acquisition and biological integration, but their comparative selectivity has not been established in head-to-head clinical studies [75,76].

Small clinical and experimental studies have reported improvements in tactile discrimination, grip-force regulation, object manipulation, and prosthetic embodiment, making these interfaces particularly attractive for applications requiring biomimetic sensory feedback. Nevertheless, their clinical translation remains limited by the need for surgical implantation, potential foreign-body reactions, fibrotic encapsulation, and uncertainties regarding long-term recording stability. These factors continue to represent major barriers to widespread clinical adoption despite their promising experimental performance [5,20,21,25,28,75].

Conversely, extraneural interfaces, including cuff electrodes and C-FINE systems, offer a more conservative surgical approach while maintaining excellent long-term stability and biocompatibility. Because these electrodes surround rather than penetrate the peripheral nerve, they substantially reduce the risk of neural injury and chronic tissue reactions. Although their lower spatial selectivity generally limits the precision of sensory restoration compared with intraneural devices, their favorable safety profile and reliable chronic performance make them particularly attractive for long-term clinical use. Recent wireless cuff-based systems further suggest that improvements in electrode design and signal processing may progressively reduce the performance gap between extraneural and penetrating interfaces [12,23,31,75].

A fundamentally different strategy involves biological, regenerative, and neuromuscular approaches, including targeted muscle reinnervation (TMR), targeted sensory reinnervation (TSR), regenerative peripheral nerve interfaces (RPNIs), and the more recently developed agonist-antagonist myoneural interface (AMI). Rather than relying exclusively on direct electrode-nerve communication, these approaches use nerve transfer, tissue reinnervation, biological signal amplification or reconstructed muscle mechanics to improve prosthetic control and sensorimotor integration. Besides improving electromyographic signal quality, biological interfaces may reduce neuroma formation, preserve physiological muscle activation, facilitate intuitive prosthetic control, and create a favorable substrate for future sensory restoration. In particular, separate early studies on RPNIs have reported long-term motor-signal stability and experimentally elicited proprioceptive or cutaneous sensations, suggesting that biological interfaces may currently represent one of the most promising strategies for long-term clinical translation [10,29,76,77,78,79].

In parallel, non-invasive sensory-feedback systems have undergone remarkable development over the last decade. Techniques based on transcutaneous electrical nerve stimulation (TENS), electrotactile stimulation, vibrotactile feedback, and mechanotactile feedback offer important practical advantages by avoiding surgical implantation while providing meaningful improvements in sensory perception and functional performance. Although these systems generally produce lower spatial resolution and less biomimetic sensory perception than implanted interfaces, they are inexpensive, easily applicable during rehabilitation, and suitable for a much broader patient population. Recent advances in biomimetic stimulation algorithms, wearable electronics, and adaptive sensory encoding have substantially improved the naturalness of the elicited sensations, making non-invasive systems increasingly attractive as either definitive rehabilitation tools or complementary solutions for patients unsuitable for implantable technologies [23,37,47,50].

Importantly, comparison among these technologies demonstrates that current neuroprosthetic research is progressively moving away from the concept of identifying a single “best” neural interface. Instead, increasing evidence supports the development of hybrid neuroprosthetic platforms capable of combining the strengths of multiple complementary technologies. Biological interfaces may provide stable long-term motor decoding, intraneural electrodes may supply highly selective sensory feedback, while non-invasive stimulation systems may enhance rehabilitation and facilitate adaptive cortical plasticity. Similarly, integration of artificial intelligence, biomimetic sensory encoding, wireless communication, and closed-loop control algorithms is expected to further improve the physiological realism of bidirectional prosthetic systems. Consequently, future clinical translation will likely depend less on the superiority of individual devices than on the successful integration of complementary neurosensory technologies within personalized bionic reconstruction strategies [23,47].

### 3.6. Clinical Considerations for Interface Selection

Interface selection should be individualized according to the level of amputation, residual nerve and muscle anatomy, functional and sensory priorities, eligibility for surgery, rehabilitation capacity, and patient preference. Direct peripheral neural interfaces may be more relevant when localized referred sensations and high spatial selectivity are prioritized, provided that the patient is eligible for invasive nerve surgery and long-term device surveillance [20,21]. Biological or neuromuscular approaches may be particularly relevant when nerve reconstruction, neuroma management, amplification of motor signals, or preservation of proprioceptive muscle relationships is also required [23]. Osseointegrated neuromusculoskeletal platforms may be considered in carefully selected patients requiring stable skeletal attachment combined with implanted control or sensory components, while accounting for surgical complexity and infection risk [8]. Non-invasive sensory-feedback or sensory-substitution systems may be preferable when surgery is contraindicated or declined, during preliminary rehabilitation and training, or when reversibility and accessibility are priorities [21]. Central stimulation remains investigational and should currently be restricted to specialized research settings. In upper-limb applications, interface selection primarily emphasizes dexterity, grip-force regulation, object discrimination, and reduction of visual dependence, whereas lower-limb applications prioritize plantar and proprioceptive feedback, gait, balance, weight transfer, and fall risk. These considerations provide a clinical decision framework rather than formal treatment recommendations because direct comparative evidence remains limited.

## 4. Discussion

Recent advances in neural engineering have profoundly transformed the field of bionic reconstruction, shifting its primary objective from simple motor replacement toward the restoration of physiological bidirectional sensorimotor communication. The studies reviewed indicate that some neural interfaces have progressed beyond initial proof-of-concept testing; however, most technologies remain investigational. The available evidence supports improvements in selected laboratory outcomes and embodiment, but consistent long-term clinical and quality-of-life benefits have not been established. However, despite remarkable technological progress, important challenges remain regarding long-term stability, standardization of clinical outcomes, scalability, and widespread clinical implementation. The following sections critically discuss the evolution of neural and related sensorimotor interfaces employed in bionic reconstruction, highlighting their mechanisms of action, clinical applications, advantages, limitations, and translational potential.

### 4.1. Evolution of Limb Neuroprosthetics: From Motor Replacement to Bidirectional Sensorimotor Restoration

The primary objective of conventional prosthetic rehabilitation has historically been the restoration of basic motor function following limb loss. Early prosthetic devices were purely mechanical systems designed to compensate for the absence of the missing limb, providing limited functionality and requiring substantial physical effort from the user [1,2,3].

The introduction of externally powered myoelectric prostheses represented a major technological milestone by enabling residual electromyographic signals to control prosthetic movements. Over the last decades, advances in pattern-recognition algorithms, multifunctional robotic hands, and intelligent control systems have substantially improved prosthetic dexterity and increased the number of controllable degrees of freedom. Nevertheless, despite these technological improvements, prosthetic control has remained fundamentally unidirectional because motor commands can be transmitted to the prosthesis without restoring physiological afferent sensory information [4,5,6].

The lack of tactile and proprioceptive feedback profoundly alters the natural sensorimotor loop. In healthy individuals, continuous bidirectional communication between the peripheral and central nervous systems enables accurate modulation of grip force, object manipulation, posture, and locomotion. Conversely, amputees using conventional prostheses rely predominantly on visual feedback, resulting in increased cognitive workload, slower motor learning, reduced embodiment, impaired motor performance, and high device rejection rates [7,17].

These limitations have progressively shifted prosthetic rehabilitation toward integrated reconstructive and neuroprosthetic approaches, which aims not only to replace the missing limb but also to restore a functional sensorimotor system through the integration of reconstructive surgery, neural engineering, implantable interfaces, and advanced robotic prostheses [4,11,13]. This multidisciplinary strategy combines targeted muscle reinnervation (TMR), targeted sensory reinnervation (TSR), regenerative peripheral nerve interfaces (RPNIs), agonist-antagonist myoneural interfaces (AMIs), osseointegrated neuromusculoskeletal prosthetic platforms, implantable electromyographic sensors, and peripheral nerve interfaces to establish increasingly intuitive communication between the patient and the prosthetic device [4,11,15,16].

Consequently, the goal of modern neuroprosthetics has evolved from restoring movement alone to recreating a physiological bidirectional sensorimotor loop capable of delivering both motor control and sensory perception. This evolution is consistent with the original principles of sensory bioengineering, which envisioned the restoration of physiological communication between biological sensory systems and artificial devices through multidisciplinary integration [9].

In this context, neural interfaces have emerged as the key enabling technology linking the peripheral nervous system with robotic prostheses. Continuous advances in electrode design, biological interfaces, biomimetic sensory encoding, wireless communication, and artificial intelligence are progressively translating these technologies from experimental proof-of-concept studies into clinically applicable neuroprosthetic systems [37,40].

The following sections review the principal categories of neural interfaces currently employed in bionic reconstruction, highlighting their mechanisms of action, clinical applications, advantages, limitations, and translational potential.

### 4.2. Taxonomy of Neural and Related Sensorimotor Interfaces

The technologies contributing to sensorimotor integration in limb prostheses operate at different anatomical and functional levels. Direct neural interfaces record from or stimulate neural tissue, whereas biological and neuromuscular approaches reorganize or amplify physiological signals without necessarily establishing direct electrode–nerve communication. Non-invasive systems provide sensory substitution through intact cutaneous pathways, while integrated prosthetic platforms combine surgical, neural, muscular, skeletal, and electronic components [4,20,23].

For this reason, the present review distinguishes six categories: (1) direct peripheral neural interfaces; (2) central neural interfaces; (3) biological or regenerative interfaces; (4) neuromuscular signal interfaces; (5) non-invasive sensory-substitution systems; and (6) integrated prosthetic platforms. This taxonomy is based primarily on anatomical target and mechanism of interaction rather than on an assumed hierarchy of clinical effectiveness [14,23].

From a neuroengineering perspective, neural interfaces may also be classified according to the level at which neural activity is acquired. Non-invasive techniques such as electroencephalography (EEG) record synchronized activity from large neuronal populations, whereas progressively more invasive approaches, including electrocorticography (ECoG), micro-electrocorticography (μECoG), penetrating microelectrode arrays, and peripheral nerve interfaces, provide increasing spatial resolution and signal specificity by reducing the distance between the recording electrode and the neural source. This hierarchical organization highlights the inherent trade-off between invasiveness, signal fidelity, anatomical coverage, and clinical applicability that underlies the design of modern neuroprosthetic systems [41].

- Extraneural Interfaces: Extraneural interfaces are positioned around the external surface of peripheral nerves without penetrating the epineurium. This configuration minimizes neural trauma and generally provides excellent long-term stability, making these devices particularly attractive for chronic clinical applications. However, because stimulation and recording occur outside the nerve fascicles, spatial selectivity is relatively limited compared with penetrating interfaces. Representative examples include cuff electrodes, Flat Interface Nerve Electrodes (FINEs), and Composite Flat Interface Nerve Electrodes (C-FINEs), which have demonstrated encouraging results in both motor decoding and sensory restoration [12,31,42].
- Intraneural Interfaces: Intraneural interfaces penetrate the nerve and establish direct contact with individual fascicles or axonal populations. This architecture enables highly selective stimulation and recording, allowing for restoration of localized tactile sensations and more physiological motor control. The most widely investigated technologies include Longitudinal Intrafascicular Electrodes (LIFEs), Transverse Intrafascicular Multichannel Electrodes (TIMEs), and Utah Slanted Electrode Arrays (USEAs). Although intraneural interfaces can provide high spatial selectivity in experimental settings, concerns remain regarding long-term biocompatibility, fibrotic encapsulation, and chronic device stability [6,7,43].
- Biological, regenerative and neuromuscular interfaces: These approaches contribute to prosthetic sensorimotor integration through distinct and non-interchangeable mechanisms. Targeted Muscle Reinnervation (TMR) primarily improves motor-signal acquisition by transferring residual motor nerves to new muscle targets. Targeted Sensory Reinnervation (TSR) redirects transected sensory nerves toward denervated skin territories, creating somatotopically organized receptive fields in which stimulation evokes sensations referred to the missing limb. Regenerative Peripheral Nerve Interfaces (RPNIs) use reinnervated free muscle grafts as biological amplifiers of peripheral nerve activity and may support motor-signal acquisition and sensory stimulation. Agonist–Antagonist Myoneural Interfaces (AMIs) preserve agonist–antagonist muscle dynamics to provide proprioceptive information and improve prosthetic joint control. Although these approaches may complement one another within integrated prosthetic systems, they differ substantially in their anatomical targets, mechanisms, primary indications, surgical requirements, and documented sensory capabilities [10,11,13,15,16].
- Non-invasive sensory-feedback systems: Non-invasive sensory-feedback systems deliver sensory information without surgical implantation through techniques such as transcutaneous electrical nerve stimulation (TENS), electrotactile stimulation, vibrotactile feedback, and mechanotactile stimulation. Although these systems generally provide lower spatial resolution than implantable neural interfaces, they offer important advantages including lower costs, minimal risks, immediate clinical applicability, and easier integration into rehabilitation programs. Recent developments in biomimetic stimulation strategies have significantly improved the quality and naturalness of the elicited sensations, making non-invasive systems an increasingly attractive alternative for selected patients [18,34,37].

Overall, each class of neural interface presents specific advantages and limitations, and no single technology currently satisfies all clinical requirements. Consequently, contemporary research is progressively moving toward hybrid strategies combining biological reconstruction, implantable neural interfaces, biomimetic sensory encoding, and intelligent decoding algorithms to maximize both motor performance and sensory restoration [10,12,25,40].

### 4.3. Current Challenges and Future Perspectives of Neurosensory Interfaces

Despite the remarkable progress achieved during the last two decades, several challenges continue to limit the widespread clinical implementation of neurosensory interfaces. One of the most important limitations identified throughout the present review is the relatively low level of clinical evidence currently available. As summarized in Table 1, most published studies consist of pilot investigations or first-in-human experiences involving only one or a few participants, frequently with heterogeneous rehabilitation protocols, different stimulation paradigms, and non-standardized outcome measures. Such methodological variability considerably limits direct comparison among technologies and prevents definitive conclusions regarding their long-term clinical superiority [10,20,21,23,25,31,50,75,78].

Another major challenge concerns the long-term reliability of implantable interfaces. Although intraneural electrodes can provide high sensory selectivity in experimental settings, chronic implantation remains associated with foreign-body reactions, fibrotic encapsulation, electrode degradation, and progressive signal instability. Conversely, extraneural electrodes generally demonstrate greater long-term biocompatibility but at the expense of reduced spatial selectivity. Biological interfaces partially overcome these limitations by exploiting physiological nerve regeneration; however, they still require technically demanding surgical procedures and highly specialized multidisciplinary rehabilitation. Consequently, improving the balance between sensory selectivity, biological integration, durability, and surgical invasiveness remains one of the principal objectives of current neuroprosthetic research [10,21,25,31].

Beyond hardware-related limitations, future clinical translation will also depend on the development of standardized evaluation protocols. Current studies employ a wide variety of clinical endpoints, including tactile discrimination, grip-force modulation, dexterity tests, embodiment questionnaires, phantom limb pain scores, gait analysis, and quality-of-life assessments. Although each parameter provides valuable information, the absence of universally accepted outcome measures complicates meta-analyses and limits the possibility of directly comparing different neural interface technologies. International consensus regarding standardized functional, neurophysiological, and patient-reported outcome measures will therefore be essential to facilitate future multicenter clinical trials and accelerate regulatory approval [20,47,50].

Future bidirectional neuroprosthetic systems are expected to increasingly rely on closed-loop architectures capable of continuously integrating neural recording, adaptive decoding algorithms, adaptive stimulation strategies, and sensory stimulation. Such systems may dynamically adjust stimulation parameters according to ongoing neural activity, thereby improving long-term stability, functional performance, and user adaptation [54,67].

Patient-specific computational modeling is also expected to play an increasingly important role by optimizing electrode positioning, predicting neural recruitment, and personalizing stimulation parameters before implantation [80]. Increasing evidence suggests that the next generation of neurosensory systems will integrate artificial intelligence, biomimetic sensory encoding, wireless fully implantable electronics, current-steering electrode technologies, and soft bioelectronic materials capable of establishing long-term, biocompatible communication with peripheral nerves. Simultaneously, advances in central neurostimulation and cortical signal decoding may further enhance the physiological integration of artificial sensory information, opening up new possibilities for personalized closed-loop neuroprosthetic systems [23,47].

Another emerging concept highlighted by recent literature is the progressive convergence of technologies that were previously considered independent. Rather than competing approaches, intraneural electrodes, biological interfaces, non-invasive sensory stimulation, osseointegrated neuromusculoskeletal platforms, and intelligent control algorithms are being increasingly combined within hybrid neuroprosthetic platforms. Such systems aim to exploit the complementary strengths of each technology by integrating stable biological interfaces, highly selective sensory stimulation, adaptive machine learning algorithms, and continuous closed-loop feedback into a single personalized prosthetic ecosystem. This integrative strategy may help address some limitations of current neurosensory interfaces, although its effects on embodiment, functional recovery, and long-term prosthesis acceptance require clinical confirmation [78,79].

Overall, the evidence reviewed suggests that the future of bionic reconstruction will not depend on the identification of a single ideal neurosensory interface but rather on the intelligent integration of complementary technologies capable of recreating increasingly physiological sensorimotor communication. Continued collaboration among surgeons, neuroscientists, engineers, rehabilitation specialists, and artificial intelligence experts will be essential to evaluate whether these technologies can be translated into clinical practice, ultimately enabling more intuitive, durable, and personalized neuroprosthetic solutions for individuals living with limb loss.

## 5. Limitations of the Review

This review has limitations related to its narrative design. Although a structured search and explicit eligibility criteria were used to improve transparency, the review was not conducted as an exhaustive systematic or scoping review. Consequently, incomplete retrieval and selection bias cannot be excluded. Publication bias and language bias also cannot be excluded, particularly because positive findings may be more likely to be published and retrieved. The marked heterogeneity of the included technologies, study designs, populations, and outcome measures also prevented quantitative synthesis and formal comparison across platforms. Review articles were used for contextual purposes and to identify additional primary publications, whereas study-specific information was checked against the corresponding primary sources whenever available. No formal certainty-of-evidence assessment was performed.

The clinical evidence is also affected by important methodological limitations. Most studies included highly selected participants and very small samples, frequently without an independent control group or blinded outcome assessment. Several investigations used repeated-measures or within-subject designs, which are vulnerable to learning, carryover, and order effects. Follow-up was often short, and many outcomes were assessed during acute or single-session laboratory testing rather than sustained home use. Selective reporting of positive functional or patient-reported outcomes cannot be excluded.

In addition, several publications originated from the same research groups and may describe overlapping participants or sequential experiments performed in the same cohorts. These reports should not be interpreted as fully independent evidence. Consequently, the number of publications may overestimate both the number of independently studied participants and the apparent consistency of the available clinical evidence. Finally, because neural-interface technology is evolving rapidly, some technological and translational conclusions may become outdated as new evidence emerges. These limitations require cautious interpretation of the comparative findings and preclude definitive conclusions regarding the superiority or routine clinical effectiveness of individual technologies.

## 6. Conclusions

Neural interfaces have enabled the generation of sensory percepts within bidirectional prosthetic systems in selected experimental and early clinical studies. The evidence reviewed in this study suggest that technological feasibility has been demonstrated across multiple platforms, including direct peripheral and central neural interfaces, biological or regenerative interfaces, neuromuscular signal interfaces, non-invasive sensory-feedback systems, and integrated prosthetic platforms, each addressing different aspects of prosthetic sensorimotor function. Recent studies have demonstrated the feasibility of eliciting tactile, proprioceptive, thermal, and multimodal percepts, with preliminary improvements in embodiment and selected laboratory outcomes in small, highly selected cohorts.

Despite these encouraging developments, clinical translation remains limited by small patient cohorts, heterogeneous methodologies, variable outcome measures, and the lack of large multicenter studies with long-term follow-up. Routine clinical adoption therefore remains limited, and the available evidence does not yet demonstrate broad improvements in long-term functional recovery, independence, prosthesis acceptance, or quality of life.

Furthermore, the comparative effectiveness, durability, long-term safety, cost-effectiveness, and scalability of most platforms remain uncertain, and no currently available technology simultaneously provides high sensory selectivity, chronic stability, minimal invasiveness, and broad clinical applicability.

Future neuroprosthetic systems may increasingly integrate biomimetic sensory encoding, multimodal feedback, adaptive neural decoding, and a deeper understanding of central neuroplastic mechanisms to recreate more complete sensorimotor loops. Continued interdisciplinary collaboration and larger longitudinal clinical studies will be required to determine whether these advances can produce sustained improvements in functional recovery and quality of life.

The future of bionic reconstruction will likely depend not on a single superior interface, but on the integration of complementary biological, neural, and computational technologies aimed at providing more personalized, adaptive, and physiologically meaningful sensorimotor communication.

## Figures and Tables

**Table 1:** Clinical studies investigating neural and related sensorimotor interfaces in limb prostheses.

Study	Interface Category	Limb	Participants	Interface	Sensory Modality/Primary Clinical Outcome	Testing Context and Follow-Up	Key Findings
**Intraneural interface**							
Rossini et al. (2010) [5]	Intraneural	Upper	1	tf-LIFEs	Bidirectional prosthetic control	Laboratory testing; 4-week implantation	Demonstrated real-time motor control and reproducible localized hand and finger sensations in one amputee; sensory-stimulation efficacy decreased after approximately 10 days.
Raspopovic et al. (2014) [6]	Intraneural	Upper	1	TIMEs	Biomimetic tactile feedback	4 weeks	Restored real-time biomimetic tactile feedback, improving object discrimination and grip-force regulation.
Oddo et al. (2016) [24]	Intraneural	Upper	1 transradial amputee; 4 able-bodied participants in complementary psychophysical experiments	TIMEs (amputee)/percutaneous intraneural microstimulation (intact subjects)	Texture discrimination	Controlled laboratory experiments	Biomimetic stimulation supported texture discrimination in one amputee; complementary neurophysiological experiments were performed in four able-bodied participants.
Valle et al. (2018) [17]	Intraneural	Upper	2	Intraneural TIMEs	Biomimetic tactile sensory feedback	Single-session laboratory evaluation	Biomimetic intraneural stimulation improved sensory naturalness and selected laboratory measures of tactile discrimination and manual performance in two participants.
Petrini et al. (2019) [25]	Intraneural	Upper	3	TIMEs	Tactile sensory feedback	Laboratory evaluations during a 6-month implantation period	Intraneural stimulation elicited tactile and proprioceptive percepts and supported selected laboratory sensorimotor tasks in three participants.
Petrini et al. (2019, Nat Med) [26]	Intraneural	Lower	2	TIMEs implanted in the tibial nerve	Sensory feedback restoration during walking	Supervised laboratory walking tests during temporary implantation	During supervised experimental walking tests, sensory feedback was associated with increased walking speed and confidence, reduced metabolic cost and fatigue, and reduced phantom limb pain.
George et al. (2019) [27]	Intraneural	Upper	1	Utah Slanted Electrode Arrays (USEAs)	Biomimetic sensory feedback	14 months	Biomimetic sensory feedback improved grip-force regulation and object discrimination in one participant during controlled laboratory tasks.
Clemente et al. (2019) [28]	Intraneural	Upper	1	Intraneural electrodes	Grip-force modulation	Laboratory testing over 2 weeks	Intraneural grip-force feedback improved grip-force control and motor coordination during repeated laboratory testing in one transradial amputee.
**Biological, regenerative, and neuromuscular interfaces**							
Clites et al. (2018) [29]	Biological	Lower	1	Agonist–Antagonist Myoneural Interface (AMI)	Restoration of proprioception during walking	Laboratory evaluation	Demonstrated physiological proprioceptive feedback and improved control of a neurally controlled lower-limb prosthesis through agonist–antagonist muscle coupling.
Vu et al. (2022) [10]	Biological	Upper	2	Regenerative Peripheral Nerve Interfaces (RPNIs)	Sensory restoration	Single session	Restoration of meaningful proprioceptive and cutaneous phantom sensations
Tan et al. (2014) [30]	Extraneural	Upper	2	Peripheral nerve cuff electrodes	Long-term sensory restoration	16–24 months	Provided stable natural touch perception for up to 24 months, improving grasp-force control, delicate object manipulation, and long-term prosthesis usability.
Charkhkar et al. (2018) [31]	Extraneural	Lower	2	High-density cuff C-FINEs	Plantar sensory feedback	Acute and repeated chronic testing	Stable localized phantom foot sensations through cuff stimulation.
Cady et al. (2025) [12]	Extraneural	Upper	2	Fully implanted wireless C-FINE and intramuscular electrode system	Fully implanted bidirectional control	4 months–2 years	First-in-human evaluation of a fully implanted wireless bidirectional neuroprosthetic platform.
**Osseointegrated neuromusculoskeletal platform**							
Ortiz-Catalan et al. (2020) [8]	Integrated prosthetic platform	Upper	4 implanted; longitudinal clinical outcomes reported for 3	Bone-anchored prosthesis with implanted neuromuscular electrodes	Long-term home prosthetic use and bidirectional control	3–7 years	Long-term home use with stable bidirectional prosthetic control was documented in three participants. Serious infection-related adverse events requiring explantation were subsequently reported in one additional participant.
**Central interface**							
Chandrasekaran et al. (2020) [32]	Spinal	Upper	4	Epidural spinal cord stimulation	Somatic sensory restoration	Up to 29 days	Restored phantom hand sensations without peripheral nerve implantation.
**Non-invasive** **sensory-feedback systems**							
Basla et al. (2022) [18]	Non-invasive sensory feedback	Lower	3 transfemoral amputees; 3 able-bodied participants for system verification	Wearable sensory leg neuroprosthesis	Somatotopic sensory feedback	Single session	Validated a wearable non-invasive sensory leg neuroprosthesis, demonstrating reliable mechanical, electrical, and functional performance while restoring somatotopic sensory feedback during lower-limb prosthesis use.
Han et al. (2023) [33]	Non-invasive sensory feedback	Upper	2 amputees + 5 able-bodied controls	Multichannel electrotactile stimulation	Wrist position and movement perception	Single session	Enabled recognition of prosthetic wrist position and movement after brief training.
Barontini et al. (2023) [34]	Non-invasive sensory feedback	Upper	5	Vibrotactile/force-feedback system	Functional manipulation	Single session	Single feedback modalities were preferred over combined feedback. Force feedback showed the greatest perceived usefulness for daily activities, although optimal feedback remained subject- and task-dependent.
Garenfeld et al. (2023) [35]	Non-invasive sensory feedback	Upper	11 (10 controls + 1 amputee)	Full-state electrotactile feedback	Closed-loop multifunctional prosthesis control	Single session	Full-state electrotactile feedback improved prosthesis position control during multi-DOF closed-loop operation.
Zhang et al. (2024) [36]	Non-invasive sensory feedback	Upper	6 participants with forearm amputation	TENS-based somatotopic sensory interface	Sensorimotor performance	Single session	Somatotopically evoked tactile sensations significantly improved prosthetic sensorimotor performance and object manipulation.
Scarpelli et al. (2024) [37]	Non-invasive sensory feedback	Upper	5 transradial amputees	Transcutaneous Electrical Nerve Stimulation (TENS)	Force and slip tactile feedback	Single session	Participants discriminated three force levels and slip directions encoded through TENS during laboratory testing.
Demofonti et al. (2025) [38]	Non-invasive sensory feedback	Lower	13 lower-limb amputees in sensory characterization; 2 completed the 4-week gait intervention	Somatotopic TENS	Gait rehabilitation	4 weeks	TENS elicited somatotopic sensations in 13 participants; selected gait and weight-distribution measures improved in the two participants who completed the 4-week intervention.
**Emerging technologies**							
Schmitt et al. (2023) [39]	Implanted peripheral sensory neuroprosthesis	Lower	1	Implanted sensory neuroprosthesis	Long-term sensorimotor integration	31 weeks	Progressive sensorimotor adaptation during prolonged home use.
Muheim et al. (2024) [40]	Sensorized thermal feedback interface	Upper	1	MiniTouch thermal feedback system	Thermal sensation	Short-term	First restoration of thermal discrimination through a prosthetic hand.

**Abbreviations:** AMI = Agonist–Antagonist Myoneural Interface; TIME = Transverse Intrafascicular Multichannel Electrode; tf-LIFE = thin-film Longitudinal Intrafascicular Electrode; RPNI = Regenerative Peripheral Nerve Interface; TENS = Transcutaneous Electrical Nerve Stimulation; C-FINE = Composite Flat Interface Nerve Electrode; EMG = Electromyography; NR = not reported, USEA = Utah Slanted Electrode Array. Participants are reported separately as amputees and able-bodied controls when applicable. Testing context and follow-up refer to the information reported in the primary publication. Some publications may describe sequential experiments or potentially overlapping participants and should therefore not be interpreted as fully independent clinical cohorts. Objective task performance, patient-reported outcomes, experimental questionnaires, and qualitative observations are reported as distinct outcome categories and should not be interpreted as equivalent evidence of functional independence or clinical effectiveness.

**Table 2:** Comparative characteristics of current neural interface technologies.

Technology	Studies	Neural Target	Application	Potential Clinical Advantages	Surgical Burden, Limitations and Evidence Gaps	Human and Home-Use Evidence
tf-LIFE/FAST-LIFE	Rossini 2010 [5]	Intrafascicular peripheral nerve	Motor decoding and sensory restoration	High neural selectivity, bidirectional communication	Invasive implantation, limited long-term evidence	One participant; laboratory testing during a 4-week implantation; no home-use evidence.
TIME	Raspopovic 2014; Petrini 2019; Clemente 2019; Valle 2018 [6,7,17,28]	Intrafascicular peripheral nerve	Biomimetic tactile feedback	Excellent spatial selectivity, natural tactile perception	Small clinical cohorts, chronic stability still under investigation	Small human cohorts; predominantly laboratory testing for up to 6 months; no established home-use evidence.
RPNIs	Vu 2022 [10]	Regenerative muscle graft	Biological motor interface with emerging sensory potential	Stable EMG signals, reduced neuroma pain, chronic performance	Surgical procedure required	Preliminary sensory evidence in two participants during single-session testing; no home-use evidence.
Targeted Sensory Reinnervation (TSR)	Gardetto et al. 2025; Serino et al. 2017 [15,16]	Reinnervated cutaneous sensory nerves	Biological sensory restoration	Physiological referred sensation, improved embodiment, intuitive tactile feedback	Requires surgical reinnervation; limited clinical evidence	Human sensory-mapping reports are available; standardized functional and sustained home-use evidence remains limited.
C-FINE cuff electrodes	Charkhkar 2018; Cady 2025 [12,31]	Peripheral nerve (extraneural)	Bidirectional prosthetic control	Good long-term stability, less invasive than intraneural electrodes	Lower fascicular selectivity	Small human cohorts; follow-up ranging from 4 months to 2 years; limited home-use evidence.
Osseointegrated neuromusculoskeletal interfaces	Ortiz-Catalan 2020 [8]	Bone-anchored implanted electrodes	Long-term prosthetic control	Stable home use, intuitive prosthesis control	Infection risk, complex surgery	Home use documented in three participants over 3–7 years; serious infection-related adverse events reported in one additional participant.
Epidural spinal cord stimulation	Chandrasekaran 2020 [32]	Dorsal spinal cord	Sensory restoration	Does not require peripheral nerve implantation	Limited long-term evidence	Four participants; laboratory testing for up to 29 days; no home-use evidence.
Electrotactile/TENS interfaces	Han 2023; Zhang 2024; Scarpelli 2024; Demofonti 2025 [33,36,37,38]	Cutaneous sensory pathways	Non-invasive sensory feedback	Completely non-invasive, easy clinical implementation	Lower spatial selectivity and less natural sensations	Small laboratory studies; limited evidence of sustained home use or benefit in activities of daily living.
Vibrotactile/haptic feedback	Barontini 2023; [34]	Skin mechanoreceptors	Sensory substitution	Simple, wearable, inexpensive	Does not restore physiological sensation	Predominantly small, single-session laboratory studies; sustained home-use evidence remains limited.
Thermal feedback systems	Muheim 2024 [40]	Integrated prosthetic sensors	Multimodal sensory feedback	Introduces thermal perception	Limited clinical evidence	Short-term laboratory testing in one participant; no home-use evidence.
Implanted lower-limb sensory neuroprosthesis	Schmitt et al. (2023) [39]	Residual peripheral sensory pathways	Long-term sensory restoration and sensorimotor integration during home use	Continuous sensory feedback, progressive embodiment, successful real-world home use	Invasive implantation; evidence limited to one participant; long-term safety and device-maintenance data remain limited.	Home use documented in one participant over 31 weeks.

**Abbreviations:** C-FINE = Composite Flat Interface Nerve Electrode; EMG = Electromyography; FAST-LIFE = Fast Longitudinal Intrafascicular Electrode; RPNI = Regenerative Peripheral Nerve Interface; TENS = Transcutaneous Electrical Nerve Stimulation; tf-LIFE = thin-film Longitudinal Intrafascicular Electrode; TIME = Transverse Intrafascicular Multichannel Electrode; TSR = Targeted Sensory Reinnervation.

## Data Availability

All data extracted and analyzed in this study are included in the article; the extraction is available from the corresponding author upon reasonable request.

## Abbreviations

The following abbreviations are used in this manuscript:

AI	Artificial Intelligence
AMI	Agonist–Antagonist Myoneural Interface
C-FINE	Composite Flat Interface Nerve Electrode
CNS	Central Nervous System
DOF	Degrees of Freedom
ECoG	Electrocorticography
EEG	Electroencephalography
EMG	Electromyography
FAST-LIFE	Fast Longitudinal Intrafascicular Electrode
FDA	Food and Drug Administration
FINE	Flat Interface Nerve Electrode
LIFE	Longitudinal Intrafascicular Electrode
MeSH	Medical Subject Heading
NR	Not Reported
RPNI	Regenerative Peripheral Nerve Interface
TENS	Transcutaneous Electrical Nerve Stimulation
tf-LIFE	Thin-Film Longitudinal Intrafascicular Electrode
TIME	Transverse Intrafascicular Multichannel Electrode
TMR	Targeted Muscle Reinnervation
TSR	Targeted Sensory Reinnervation
USEA	Utah Slanted Electrode Array
μECoG	Micro-Electrocorticography
